# Effect of Antibacterial Silver-Releasing Filler on the Physicochemical Properties of Poly(Methyl Methacrylate) Denture Base Material

**DOI:** 10.3390/ma12244146

**Published:** 2019-12-11

**Authors:** Grzegorz Chladek, Katarzyna Pakieła, Wojciech Pakieła, Jarosław Żmudzki, Marcin Adamiak, Cezary Krawczyk

**Affiliations:** 1 Faculty of Mechanical Engineering, Silesian University of Technology, ul. Konarskiego 18a, 44-100 Gliwice, Poland; katarzyna.pakiela@polsl.pl (K.P.); wojciech.pakiela@polsl.pl (W.P.); Jaroslaw.Zmudzki@polsl.pl (J.Ż.); marcin.adamiak@polsl.pl (M.A.); 2 Department of Dental Technology, Medical College, ul. 3 Maja 63, 41-800 Zabrze, Poland; crkrawczyk@interia.pl

**Keywords:** polymethyl methacrylate, denture, antibacterial properties, silver, mechanical properties, sorption, solubility, wear resistance

## Abstract

Colonization of polymeric dental prosthetic materials by yeast-like fungi and the association of these microorganisms with complications occurring during prosthetic treatment are important clinical problems. In previously presented research, submicron inorganic particles of silver sodium hydrogen zirconium phosphate (S–P) were introduced into poly(methyl methacrylate) (PMMA) denture base material which allowed for obtaining the antimicrobial effect during a 90 day experiment. The aim of the present study was to investigate the flexural strength, impact strength, hardness, wear resistance, sorption, and solubility during three months of storage in distilled water. With increasing S–P concentration after 2 days of conditioning in distilled water, reduced values of flexural strength (107–72 MPa), impact strength (18.4–5.5 MPa) as well as enhanced solubility (0.95–1.49 µg/mm^3^) were registered, but they were at acceptable levels, and the sorption was stable. Favorable changes included increased hardness (198–238 MPa), flexural modulus (2.9–3.3 GPa), and decreased volume loss during wear test (2.9–0.2 mm^3^). The percentage changes of the analyzed properties during the 90 days of storage in distilled water were similar for all materials.

## 1. Introduction

In the United States, only 34% of adults aged 40–64 have retained all of their permanent teeth, and nearly 19% of patients aged 65 and over suffer from edentulism [1]. Similarly, 16.3% of Indians and almost 22% of Mexicans aged 50 and above are edentulous [2]. These data show how common the problem of missing teeth is in modern society, regardless of race or region of the world. Many patients are users of conventional removable dentures, mainly made of polymethyl methacrylate (PMMA) due to the fact that they allow for obtaining products at an affordable price, in comparison to, for example, implant-fixed dentures [3]. In addition, they are characterized by acceptable quality in terms of improving oral function, enhancing phonetics, facilitating social engagement, and aesthetics [4].

Colonization of polymeric dental prosthetic materials by *Candida* species and the association of these microorganisms with complications, such as denture stomatitis occurring during prosthetic treatment, is an important clinical problem which has been widely described in the literature [5,6,7]. Fungi and bacteria occurring in the mouth are the sources of many ailments and systemic diseases including heart, circulatory system, kidney, stomach or esophagus problems [8,9]; therefore, poor microbiological status of dentures can contribute to the deterioration of overall health [10]. Humid microenvironment under prostheses and decreased possibilities of mucosal self-cleaning by saliva promote growth of microorganisms [6,11], and only a few dozen minutes after cleaning, the denture surface begins to be re-colonized by bacteria and fungi [12,13].

In order to reduce the indicated problems, various strategies are proposed. Antifungal drugs, such as nystatin or amphotericin B, can eliminate pathogenic microorganisms from the surface of tissues [14] and have also been added experimentally to PMMA material [15]. However, *Candida albicans* show increasing resistance during treatment of oral fungal infections [16].

Numerous studies have proved the varied effectiveness of removing microorganisms from the surface of prosthetic materials, for example, by using chlorhexidine gluconate, guanidine solution, peroxides, irradiation microwaves, or buy brushing with toothpaste, but these methods result in the loss of various functional properties, including increased roughness [17,18,19,20,21,22], which can facilitate the recolonization of prosthetic materials [23]. It should be emphasized that there are studies questioning the possibility of fully effective removal of microorganisms from the denture using mechanical or chemical cleaning methods [24], suggesting the possibility of penetration of *C. albicans* into the interior of acrylic materials [25,26,27] which indicates limited disinfection possibilities. Due to the problems associated with colonization by *Candida* species, investigations related with the development of new materials are being conducted in two directions: the introduction of additional monomers with antimicrobial properties and the manufacturing of composites by introducing the fillers with antimicrobial properties [28]. Such materials would be characterized by both increased resistance to microbial colonization and support for the treatment of, for example, *Candida-*infected mucosa. Antimicrobial efficacy in vitro has been confirmed so far in laboratory experiments conducted with PMMA denture base materials modified with numerous metal and metal oxides nanoparticles such as ZrO_2_ [29], TiO_2_ [30,31], ZnO [32], platinum [33], silver [34,35] or silver microparticles [36]. Silver nanoparticles have particularly strong antimicrobial properties; however, studies have simultaneously shown that the introduction of this type of additive to prosthetic materials causes an intense brown color of PMMA resin due to the plasmon effect [35] which is unacceptable for aesthetic reasons. Despite these problems, materials containing silver are still considered an attractive antimicrobial additive; therefore, in the previously presented research, it was proposed to introduce submicron inorganic particles of silver sodium hydrogen zirconium phosphate (S–P) as an antimicrobial additive to PMMA [37]. This white filler does not cause the initial dark coloring of modified materials.

In the published first part of our investigations, the morphology and antimicrobial properties of the developed composites were investigated [37]. Most of the experimental materials presented efficacy against *C. albicans*, even when samples were stored in distilled water for a three-month period. However, the morphologies of the composites were not homogenous. This indicated a risk of unfavorable changes in physicochemical properties.

Appropriate mechanical properties and their stability are particularly important for the functioning of complete and partial dentures. Denture-based materials must show strength, ensuring long-term functioning of the prosthesis loaded with functional and parafunctional masticatory forces [38]. Even 68% of the mentioned types of prostheses are damaged during the first few years after manufacturing which clearly shows the scale of this clinical problem and the importance of mechanical properties [39]. The denture fracture may result from flexural forces due to, for example, the improper fabrication, poor fit or lack of balanced occlusion [40]. Moreover, most PMMA partial or complete dentures are removable; thus, their resistance to shock-induced fractures, represented by impact strength, is no less important due to the possibility of their falling or being damaged during the action of violent forces caused by other events [41]. Between 39.5% and 56% of fractures occur as a result of the fall of dentures [41,42]. Other material properties that affect the durability of PMMA dentures are their hardness and wear resistance which determine the surface conditions of prosthetic materials [43,44]. The significance of the abovementioned properties is demonstrated by a significant number of studies on their changes in the context of various aspects of the functioning of materials such as the use of cleaning agents or the consumption of hot/cold foods and drinks (thermocycling) [22,45]. Considering that these materials function in an environment with 100% humidity, their behavior under these conditions is equally important, not only in the context of mechanical/tribological properties, but also due to the fact of water absorption and release of material components into the environment. Therefore, the aim of this paper was to investigate the effect of the introduction of S–P on the mechanical properties, wear resistance, and the sorption and solubility of the modified PMMA denture base material during three months of storage in distilled water. Our hypothesis was that composites filled with silver sodium hydrogen zirconium phosphate would show physicochemical properties relevant to the application being considered.

## 2. Materials and Methods

### 2.1. Material and Sample Preparation

#### 2.1.1. Material Preparation

The method of the composites’ preparation was described in a previously published work [37]. The PMMA heat-cured denture base resin, Meliodent Heat Cure (Heraeus Kulzer, Hanau, Germany), was used as a matrix and silver sodium hydrogen zirconium phosphate (Milliken Chemical, Spartanburg, SC, USA) as filler. To exclude sedimentation during material storage, the filler was added only to the pre-polymerized “powder” component of the “*powder–liquid*” system. The components were mixed using a planetary ball mill (Pulverisette 5, Fritsch, Idar–Oberstein, Germany) with 50 ZrO_2_ balls with a diameter of 10 mm. A milling time of 5 min with a frequency of rotation of 400 rpm was used. The mass of modified components (PMMA with S-P) obtained during one milling process was from 10–10.4 g). During the materials’ preparation, we used the AS 110/C/2 analytic scale (Radwag, Radom, Poland) with a measurement accuracy of 0.1 mg, but the real mass of the S–P mixed with PMMA powder was determined by the possibility of manual dosing, and the error did not exceed 0.01%. Moreover, the concentrations of S–P in liquid, listed in Table 1, are only theoretical. Dosing of the liquid component was possible with an accuracy of one drop (0.01–0.015 g), so the error mainly depended on the mass of the used liquid necessary for the particular samples’ preparation in flasks and was not higher than 0.05%. However, during cross-linking, many necessary activities must be performed (mixing, packing into flask, etc.) and some mass of the monomer evaporates and, in fact, those considerations have only limited practical value. The list of the obtained materials with filler concentrations is presented in Table 1. It should be noted that the filler was mixed with PMMA powder, but after polymerization of the samples, the filler was present in areas of materials formed during polymerization from the “liquid” component (mainly methyl methacrylate). Samples were polymerized in accordance with the instructions of the resin manufacturer.

#### 2.1.2. Sample Preparation

**Figure 1:**
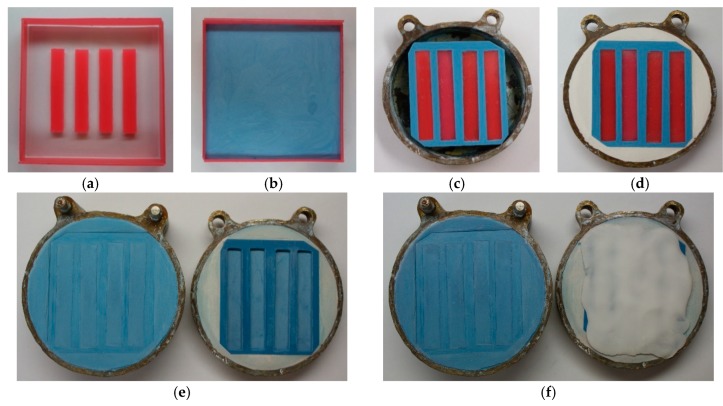
The stages of mold manufacturing for sample preparation: models of the samples before (**a**) and after (**b**) filling with dental stone; mold with models of samples (**c**); mold mounted in the first part of flask (**d**); ready mold in the flask (**e**), and “packing” of the material during the curing process (**f**).

The samples for most tests (i.e., flexural properties, impact strength, hardness, wear resistance) were prepared using a standard flasking technique used in dental prosthetics to maintain the typical polymerization conditions provided by the manufacturer of the PMMA resin. The most important stages of the procedure are related with mold preparation and are shown in Figure 1. First, the prepared models of the samples were filled with dental stone (gypsum type III, Stodent III, Zhermack, Badia Polesine, Italy) (Figure 1a–b) to create the mold. After setting of the dental stone, the mold was trimmed to obtain the required external dimension, determined by the internal dimensions of the flask (Figure 1c). Next, each mold was placed in the first part of flask and mounted using model plaster (gypsum type II, Stodent II, Zhermack, Badia Polesine, Italy) (Figure 1d). After setting, the dental plaster separating medium (Isofix 2000, Renfert GmbH, Hilzingen, Germany) was applied. Next, the second part of the flask was mounted and dental stone was poured to about 1/3 of its height. After setting, the dental plaster was used to fill the flask and flask was closed with a cover. After setting of the dental plaster, the models of the samples were removed and the molds (Figure 1e) were covered with separating media (Izolit SL, Chema-Elektromet, Rzeszów, Poland) before PMMA material and composite packing (Figure 1f). The samples of all materials were cured in accordance with the instructions of the manufacturer of the used resin.

After curing, the samples were taken out of the mold, the excess of material was cut off, and the specimens were then wet-ground (Labo-Pol25, Struers, Willich, Germany) with P220 and, finally, P500-grit abrasive paper and thoroughly rinsed in water. Samples were divided into five groups (storing conditions), and one sample per group was prepared from one powder + liquid mixture. The groups of samples were stored in distilled water at 37 ± 1 °C for 2 days ± 2 h, 7 days ± 2 h, 30 days ± 2 h, 60 days ± 2 h, and 90 days ± 2 h. Potential detailed procedures for sample preparation and sample dimensions for specific tests are provided in the descriptions of the test methods.

Samples for sorption and solubility tests were cured in stainless steel molds in accordance with the ISO standard [46]. The use of this technique was justified by the restrictive requirements of the sample dimensions and sample purity (elimination of separating medium and gypsum). A 23 µm thick polyester film (DuPont Teijin Films, Chester, USA) was used as a spacer to prevent sticking of parts of molds/adhesion of materials during the curing process [46].

### 2.2. Methods

#### 2.2.1. Flexural Properties

A three-point bending test was carried out using a universal testing machine (Zwick Z020, Zwick GmbH & Com, Ulm, Germany) based on the ISO 20795-1:2013-07 standard [46] with some modifications, i.e., additional storing times in the water were used. Specimens measuring 65 mm × 10 mm × 3.3 mm were prepared using a method described in Section 2.1.2. Twenty-five samples were prepared from each material. After conditioning, the specimen was removed from the water, placed on supports, and the test was performed at a cross-head speed of 5 mm/min. The distance between the supports was 50 mm. Flexural strength and flexural modulus were calculated according to the equations:

(1) $$\sigma =\frac{3Fl}{2bh^{2}}$$

(2) $$E=\frac{F_{1}l^{3}}{4bh^{3}d}$$

where *σ* is the flexural strength (MPa); *E* is the flexural modulus (GPa); *l* is the distance among the supports (mm); *b* and *h* are the specimen’s width and height (mm); *F* is the maximal force (N); *F_1_* is the load at a chosen point at the elastic region of the stress–strain plot (kN); and *d* is the deflection at *F_1_* (mm).

#### 2.2.2. Impact Strength

The Charpy impact strength test was conducted in accordance with the ISO 179-1:2010 standard [47] on a pendulum impact tester (HIT 25, Zwick GmbH & Com, Ulm, Germany). The unnotched specimens measuring 80 mm × 10 mm × 4 mm were prepared as described in Section 2.1.2, and, after conditioning, the specimen was removed from water, placed on supports, and the test was performed. The distance among the supports was 62 mm. The following formula was applied to calculate impact strength:

(3) $$a_{cU}=\frac{E}{b\times d}\times 10^{3}$$

where *a_cU_* is the energy absorbed by breaking the test specimen (J); *b* and *d* are the width and thickness of the specimen, respectively (mm).

#### 2.2.3. Fracture Analysis

Scanning electron microscopy, on a Zeiss SUPRA 35 (Zeiss, Oberkochen, Germany), was used to characterize the fractures of the samples broken by the impact and the three-point bending tests. All specimens were sputtered with gold before observations. Compact and smooth surface fields present brittle fracture modes, while a rough and jagged appearance presents intermediate (brittle to ductile) fracture modes [48,49]. Particular attention was paid to the impact of filler presence and aggregation on the appearance of fractures. If the fragments of fractured specimens could be repositioned at the fractured line presenting a smooth surface, the fractures were classified as brittle. Conversely, those presenting plastic deformation, exhibiting rough and jagged surfaces, were recorded as ductile [12]. Observations were performed at accelerating voltages from 5 to 10 kV.

#### 2.2.4. Hardness

The ball indentation hardness (*H*) was determined according to the ISO 2039-1 standard [50] on the Zwick 3106 hardness tester (Zwick GmbH & Com, Ulm, Germany). The specimens measuring 65 mm × 65 mm × 4 mm were prepared as described in Section 2.1.2. Four samples for each material/storing time were prepared, and three indentations were made on each sample. A steel ball, 5 mm in diameter, was indented into the materials. A test load of 358 N was applied for 30 seconds. The ball indentation hardness (*H*) was calculated according to the following equation:

(4) $$H=\;F_{m}\times \frac{\left(\frac{0.21}{h-0.25+0.21}\right)}{\pi dh_{r}}\;$$

where, *H* is the ball indentation hardness (MPa); *h* is the depth of impression after correcting for the deformation of the frame (mm); *d* is the diameter of the ball indenter (mm); *F_m_* is the test load on the indenter (N).

#### 2.2.5. Wear Resistance

The samples for tribological tests measuring 30 mm × 30 mm × 10 mm were prepared as described in 2.1.2. The experiment was performed on a CSM Tribometer (CSM Instruments, Peseux, Switzerland) using the "ball on disc" method based on the methodology presented in the standards ASTM G99-95A [51] and ISO/TS 14569-2:2001 [52]. During wear testing the specimens were subjected to rotational motion and were kept in permanent contact with the spherical antagonist – a Al_2_O_3_ ball, 6 mm in diameter (Gewa, Zabrze, Poland). The experiment was conducted in distilled water at 37 ± 1 °C. The normal force applied by weight was 10 N, the relative sliding velocity was kept constant at 10 mm/s, the diameter at which the track was made was 4 mm, the total sliding distance was 100 m. After tests for each sample the profile of the cross-sectional area of wear track was measured at 6 points spaced about 60° apart (TalyProfile Lite, Tylor-Hubson, Leicester, U K) the mean area of profile was determined and finally the volume loss (mm^3^) was calculated. Scanning electron microscopy, on a Zeiss SUPRA 35 (Zeiss, Oberkochen, Germany), was used to characterize traces left after wear tests. Observations were performed at accelerating voltages 5 kV.

#### 2.2.6. Sorption and Solubility

Sorption and solubility were tested using the method presented in the ISO standard [46]. Five test samples of each material, measuring 50 mm in diameter and 0.5 mm in thickness, were cured in stainless steel molds. The samples were dried inside desiccators with freshly dried silica gel at 37 ± 1 °C and weighed daily (Analytic Scale AS/X, Radwag, Radom, Poland) until changes in mass were no higher than 0.2 mg (mass recorded as *m_1_*). The thickness and diameter of the samples were measured with a digital caliper with an accuracy of 0.01 mm. Samples were placed in distilled water at 37 ± 1 °C for 7 days and weighed after storing (mass *m_2_*). After that, the drying process, as described above, was repeated, and the stable mass was denoted as *m_3_*. Sorption and solubility were calculated using the following equations:

(5) $$w_{sp}=\frac{m_{2}-\;m_{3}}{V}$$

(6) $$w_{sl}=\frac{m_{1}-\;m_{3}}{V}$$

where *w_sp_* is the sorption, w_sl_ is the solubility, *m_l_* is the initial mass of the dried sample (µg); *m_2_* is the mass after storing (µg), *m_3_* is the mass after the second drying (µg), and *V* is the volume of the sample, (mm^3^).

#### 2.2.7. Statistical Analyses

Statistical analysis of the results was performed with the use of the Statistica 13.1 software (TIBCO Software Inc., Palo Alto, CA, USA). The distributions of the residuals were tested with the Shapiro–Wilk test, and the equality of variances was tested with Bartlett test. When the distribution of the residuals was normal and the variances were equal, the one-way or two-way ANOVA with Tukey’s HSD post-hoc tests were used (α = 0.05), otherwise the non-parametric Kruskal–Wallis test (α = 0.05) was used.

## 3. Results

### 3.1. Flexural Properties

**Figure 2:**
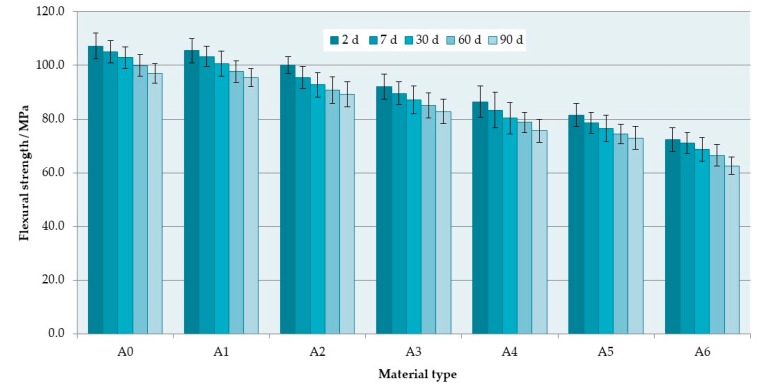
Mean flexural strength values with standard deviations.

The mean flexural strength values are presented in Figure 2. The S–P concentration significantly decreased the flexural strength values of the composites (Table 2). The mean values after 2 days of conditioning in distilled water were 107.2 MPa for the A0 material and 72.4 MPa for the A6 composite (a reduction of 32%). Storing time also had a significant influence (*p* < 0.0001) on the hardness values. The mean values decreased after 90 days and were 96.9 MPa for A0 and 62.6 MPa for A6. For all materials, similar reductions in the flexural strength values were noted (from 9% to 12%).

**Figure 3:**
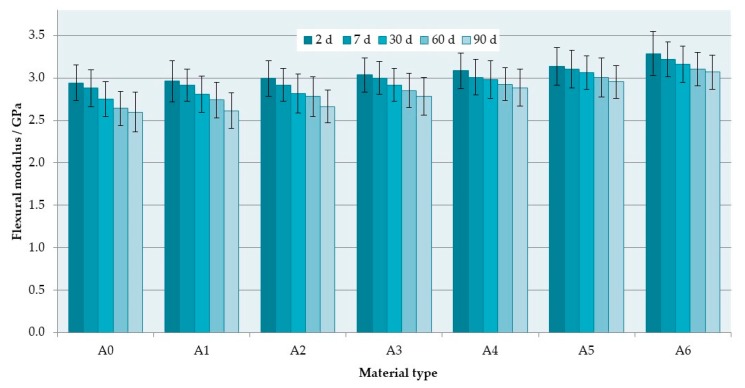
Mean flexural modulus values with standard deviations; the different uppercase letters (A–D) for each storing time show significantly different results at the *p* < 0.05 level.

The flexural modulus (Figure 3) increased with increasing S–P concentration; however, the changes were statistically significant (Table 3) only for three longer storing times. The highest increase in flexural modulus values between A0 and A6 materials was 0.5 GPa (after 90 days). The storing time had no statistically significant influence (*p* > 0.05) on the flexural modulus values, but they were noticeably reduced from 0.18 GPa to 0.35 GPa.

**Figure 4:**
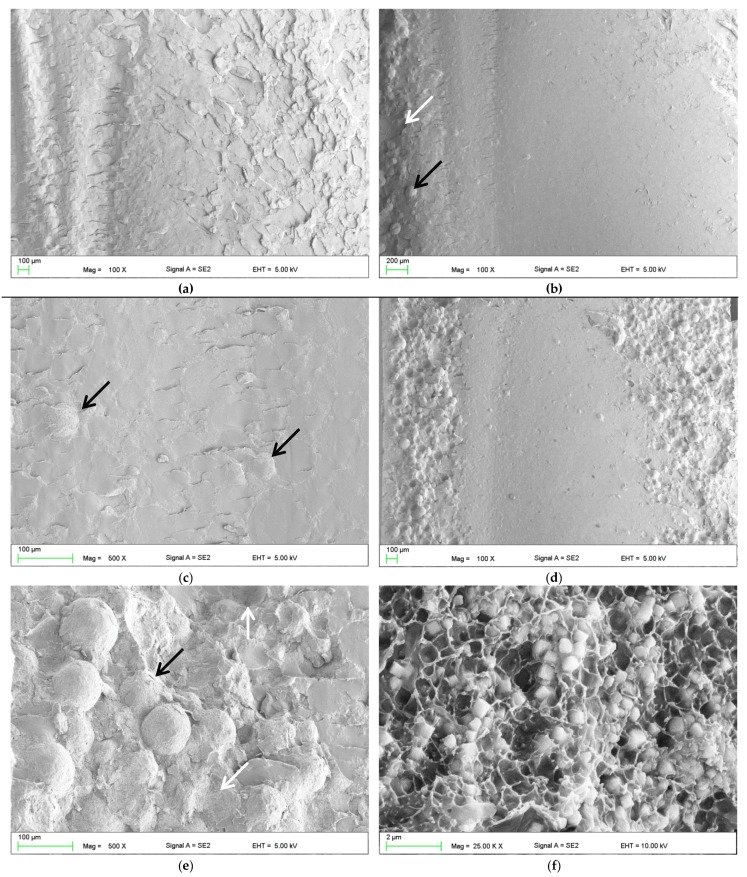
Representative SEM images presenting the fracture surfaces of flexural test specimens. Control (A0) (**a**). composites with antimicrobial filler concentrations of 1% (**b**,**c**) and 4% (**d**–**f**). Black arrows indicate the areas determined by the shape of poly(methyl methacrylate) (PMMA) pre-polymerized spheres; white arrows indicate the spherical niches remaining after them.

The SEM observations of fractures showed that the morphology of the deformed regions changed with increasing S–P concentration. For the control material (Figure 4a), areas representing a brittle fracture mode (i.e., smooth and compact surface) and an intermediate form of fracture (i.e., jagged and rough appearance) were observed. For the obtained composites, a brittle fracture mode (smooth surface) was clearly visible in the central part of the fractures, but near the lower and upper edge of the samples, there were characteristic areas showing the presence of spherical shapes determined by the shape of PMMA pre-polymerized particles of the “powder” component (Figure 4b–e), the examples of which are indicated by black and white arrows. The area of this type of morphology increased with the increasing concentration of the filler (please compare Figure 4b,d). In Figure 4f, it is shown the presence in spherical structures of a large number of cubic filler particles and the spaces formed after pulling out S–P particles from the matrix.

### 3.2. Impact Strength

**Figure 5:**
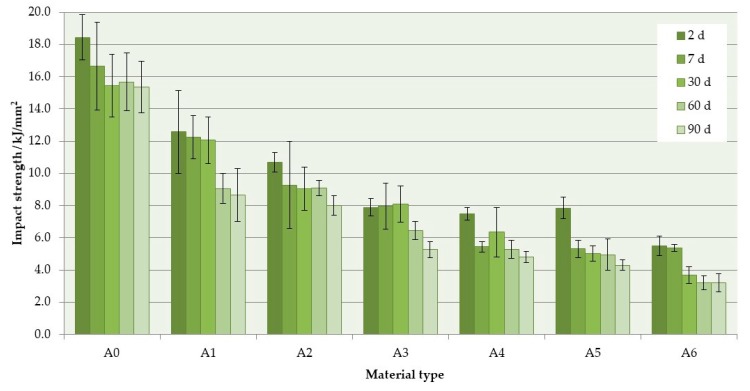
Mean impact strength values with standard deviations. The different uppercase letters (A–D) for each storing time and lowercase letters (a–c) for each material show significantly different results at the *p* < 0.05 level.

The mean impact strength values are presented in Figure 5. The S–P concentration significantly decreased the impact strength values of the composites (Table 4). The mean values after 2 days of conditioning in distilled water were 18.4 kJ/mm2 for A0 material and 5.6 kJ/mm^2^ for A6 composite (a reduction of 70%). Storing in distilled water also caused a reduction of mean values of impact strength, and, for four out of six materials, it was statistically significant (*p* < 0.05). After 90 days, the reduction was 17% for control material, whereas for the A6 composite, it was 42%.

**Figure 6:**
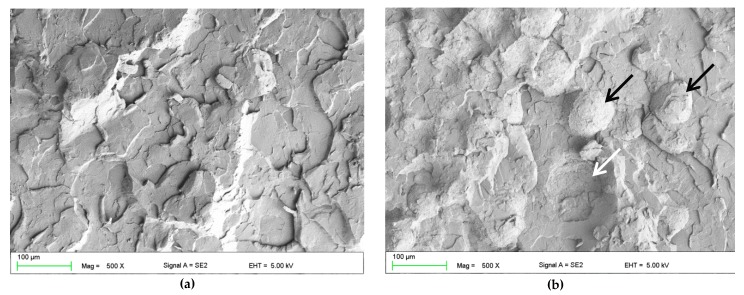
Representative SEM images presenting the fracture surfaces of impact strength test specimens. Control (A0) (**a**). Composites with an antimicrobial filler concentration of 4% (**b**). Black arrows indicate the areas determined by the shape of PMMA pre-polymerized spheres; white arrows indicate the spherical niches remaining after them.

The SEM observations (Figure 6) of impact fractures showed that the morphology where a rough and jagged appearance presents an intermediate fracture mode (brittle to ductile) was, in general, similar for all materials. However, the areas showing the presence of spherical shapes, determined by the shape of PMMA pre-polymerized particles (Figure 6b) were present, and their number increased with the increasing concentration of the filler. In comparison to the morphologies of fracture after flexural tests, these structures were observed much less frequently for the same filler concentrations, and the tendency to create areas of this type near the upper/lower surface of the samples was not clearly demonstrated.

### 3.3. Hardness

**Figure 7:**
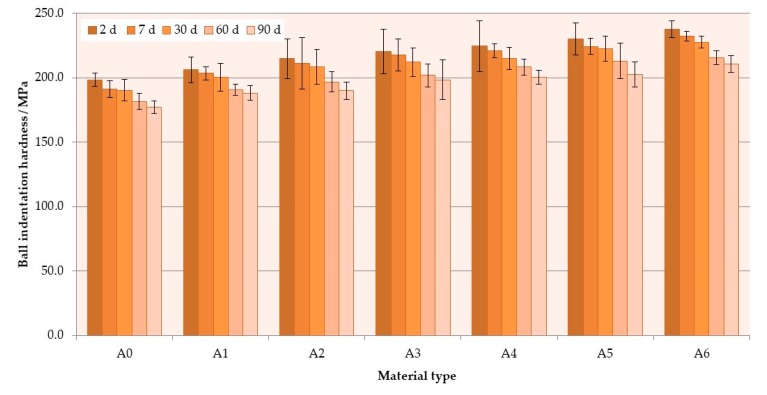
Mean ball indentation hardness values with standard deviations. The different uppercase letters (A–E) for each storing time and lowercase letters (a–c) for each material show significantly different results at the *p* < 0.05 level.

The mean hardness values are presented in Figure 7. The hardness values significantly (Table 5) increased with the antimicrobial filler concentration. The mean hardness values after 2 days of conditioning in distilled water were 192 N/mm^2^ for A0 and 232 N/mm^2^ for A6 composite. Storing time also had a significant influence (*p* < 0.0001) on the hardness values. The hardness values decreased with storing time. After 90 days, the mean values for the A0 and A6 materials were 177 N/mm^2^ and 211 N/mm^2^, respectively. The reduction of hardness values was from 9% to 12%; thus, it was similar for all materials.

### 3.4. Wear Resistance

**Figure 8:**
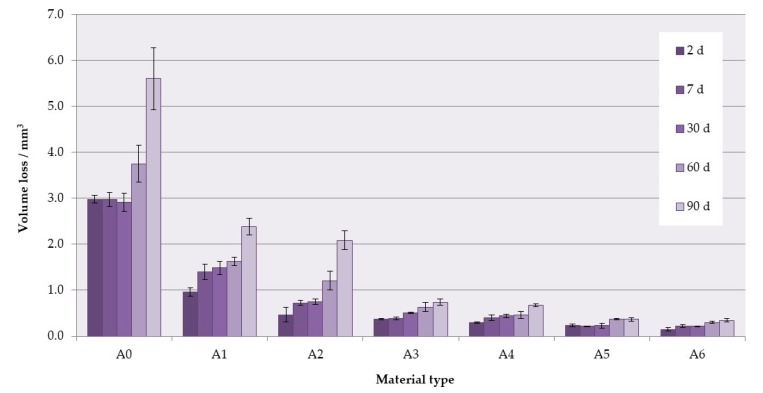
Mean volume loss values with standard deviations. The different uppercase letters (A–E) for each storing time and lowercase letters (a–c) for each material show significantly different results at the *p* < 0.05 level.

The mean values of volume loss after wear tests are presented in Figure 8. For higher S–P concentrations, significantly decreased volume losses were recorded (Table 6), and increased storing time caused a significant increase of volume loss values (*p* < 0.05). The mean values after 2 days of conditioning in distilled water were 2.98 mm^3^ for the A0 material and 0.15 mm^3^ for the A6 composite (a reduction of 95%). After 90 days, volume losses were 5.6 mm^3^ for A0 and 0.35 mm^3^ for the A6 composite.

**Figure 9:**
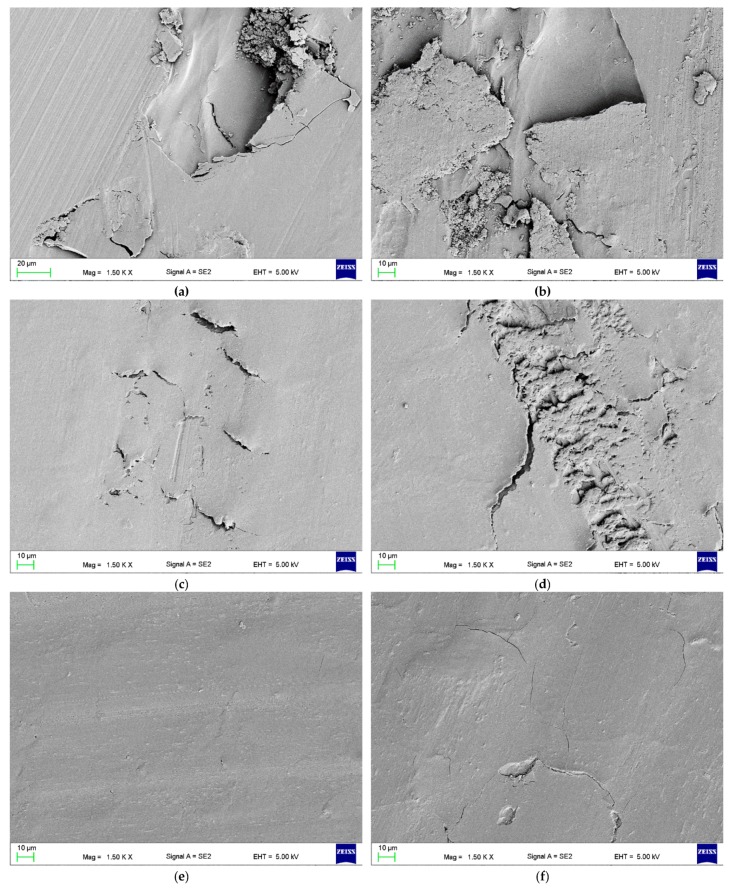
Representative SEM images presenting the traces left after wear tests on the samples of materials A0 (**a**,**b**), A3 (**c**,**d**), and A5 (**e**,**f**) stored in distilled water for 2 days (**a**,**c**,**e**) and 90 days (**b**,**d**,**f**).

The representative SEM images of the traces left after the wear test showed that the intensity of the occurrence of wear mechanisms was associated with the mass concentration of the S–P and the conditioning time. The dominant feature was abrasive wear as a result of plowing. There were numerous scratches and micro-craters parallel to the direction of counter-sample movement on the surface of the traces, and their number and depth decreased with the increase of the filler content (Figure 9a–d). For the materials from A0 to A3, the areas of surface delamination were noted. On the wear traces for materials from A0 to A4, the areas indicating plastic deformation and fatigue wear mechanism were observed. For samples of the A5 composite stored up to 30 days, only uniform abrasion was observed with slight surface scratches in the direction of the movements of the antagonist which indicated the appearance of plowing (Figure 9e). A similar situation was observed for the A6 composite (all storing times). For the A5 composites conditioned from 60 to 90 days, traces of fatigue wear and microcracks were observed over the entire surface of the wear track (Figure 9f).

### 3.5. Sorption and Solubility

**Figure 10:**
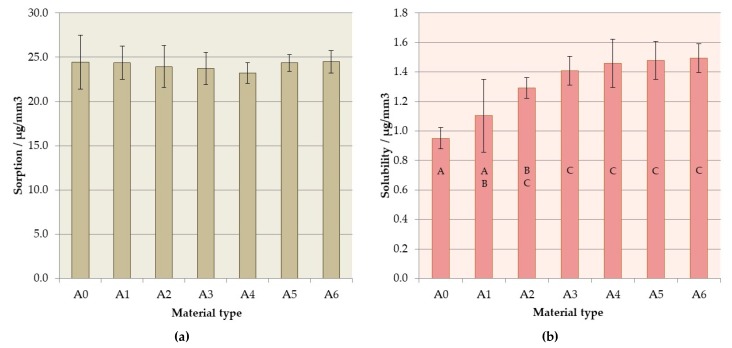
Mean and standard deviations of (**a**) sorption and (**b**) solubility. Different lowercase letters (a–c) show significantly different results at the *p* < 0.05 level.

There were no statistically significant differences between mean sorption values (*p* = 0.9248), presented in Figure 10a, which fell within the range of 23.20 µg/mm^3^ (A4) to 24.49 µg/mm^3^ (A6). The obtained values for all samples of each tested material were below the maximum limit of 32 µg/mm^3^ allowed by the ISO standard [46].

The solubility values (Figure 10b) increased with increasing S–P concentration (*p* < 0.0001); however, the changes were not statistically significant (*p* > 0.05) for materials from A2 to A6. Excluding one sample of A6 composite, all the obtained sorption values were below the maximum limit of 1.6 µg/mm^3^, so all materials were within the limit [46].

## 4. Discussion

The paper presents the results of the second stage of research on the influence of S–P introduction as an antimicrobial filler on the properties of PMMA denture base material. In the previously published part [37], the antimicrobial properties were confirmed via three-month in vitro experiments; thus, further tests were needed to investigate the other properties of the obtained composites related to their application. Additionally, S–P has also been investigated in our other works as an additive into silicone soft lining material [53] and direct restorative photopolymerizable resin-based composites [54], where only a slight influence of the filler on some physicochemical properties was noted. However, for the currently tested composites, the matrix and the method of introducing the filler into it were different which determined the inhomogeneous morphology of polymerized composites related with the used components (PMMA pre-polymerized particles) [37]. This could affect the physicochemical properties, thus investigations were conducted.

During most tests (excluding sorption and solubility), the conditioning of the samples for 90 days was conducted. The period of the samples’ storing was based on the literature. Although studies involving tests of mechanical properties of materials aged in clinical conditions are rare, it is proven that the use of dentures in the oral cavity by patients for a period of 2 to 10 years is the cause of the deterioration of acrylate materials’ hardness [55]. However, the dynamics of this process under clinical conditions was not tracked, because it requires obtaining the samples and conducting material tests at specific time intervals. This is one of the reasons why laboratory tests are performed much more frequently in this regard. These tests are usually conducted for 60 to 120 days, and the results have shown that mechanical properties of denture polymers reach equilibrium after up to 4 months [56], but most often for modern materials, this period does not exceed 60 days [57,58]. For this reason, the duration of the experiment in this study was limited to 90 days, and, after 60 days, no statistically significant changes in the mechanical properties of the materials were found, although insignificant changes were still visible.

During conditioning, the choice of medium may also influence the results. Typical media used are water and artificial saliva; however, using water is recommended by the ISO 20795-1:2013-07 standard for testing the mechanical properties of denture base materials [46]. Moreover, investigations indicate that when distilled water is used, the values of mechanical properties are lower and liquid absorption is higher than after storing in artificial saliva, even if these differences are not statistically significant [58,59]. This shows that using distilled water in these types of experiments was a rational choice, because water has, at least, the same degrading effect in comparison to artificial saliva. In addition, the use of water is justified in the first stages of research for practical reasons, because it allows creating very repetitive conditions. However, it should be noted that both mentioned liquids (i.e., water and artificial saliva) do not fully reflect real conditions, because salivary enzymes may also be the cause of polymer degradation and, as a consequence, lead to a reduction in surface hardness or wear resistance [60]. Moreover, Miranda et al. [61] suggested that liquids with lowered pH values (higher acidity) may influence the polymeric matrix of the resin by reacting with ester groups from acrylates which can create molecules of alcohol and carboxylic acid. This may lower the pH value inside the resin matrix and accelerate the degradation of the materials which should also be considered in the context of the use of dental prostheses.

The flexural strength and modulus using three-point bending tests are mechanical properties with limits that are specified by the ISO 20795-1:2013-07 standard for denture base materials, and they are related with the behavior of materials under clinical conditions. The PMMA resins during service in the mouth are subject to flexural fatigue as the denture base undergoes repeated masticatory loading [62]; thus, the high flexural strength is considered essential to denture durability, especially when gradual and irregular alveolar absorption processes cause tissue-borne dentures to be unevenly supported [63,64] but also when a perfect fit of a denture to the well-developed convex residual ridges occurs, because the denture is lifted at the non-working-side [65,66]. Flexural modulus influences denture stiffness. Their lower values are favorable in increasing the absorbed energy before fracture of the denture base, but a higher flexural modulus is recognized as clinically advantageous [67]. Partial dentures made of materials with a lower modulus of elasticity are more easily deformed during chewing and, as a result of which, locally higher loads can be transferred to the mucosa under the prosthesis [68]. Therefore, the use of materials with a lower flexural modulus may be the reason for increased pain associated with the increase in mobility of the dentures and their worse stabilization which can be the cause of the recorded decrease in chewing efficiency [69]. The increase in the antimicrobial filler mass concentration resulted in reduced flexural strength but, on the other hand, caused an increase in flexural modulus values. For all composites, the obtained values were higher than the indicated minimum (flexural strength −65 MPa, flexural modulus −2 GPa). Moreover, the registered flexural strength and modulus values for commercially available hot polymerized prosthetic PMMA resins ranged from 60 to 120 MPa [48,70,71,72,73], so it is the range analogous to that obtained for all the considered experimental composites. The flexural modulus values for heat-cured acrylic denture base resins reported in literature ranged from 2.1 to 3.1 GPa [48,74] which means that the analyzed materials are comparable in this respect with the best commercially available resins. The decrease in flexural strength and modulus values during conditioning in distilled water is a typical process noted for acrylates and related to the interaction of the liquid with the polymer matrix [75,76]. This is usually linked with the plasticizing effect exerted by water molecules penetrating into the materials [48], and this process is partially reversible [77]; however, other changes related to the release of components into the environment and the degradation of acrylates due to the hydrolysis are irreversible [78]. The percentage of reduction was the same regardless of filler content which indicates that this process was not determined by the filler content but by the properties of the polymer matrix.

Another important mechanical property of prosthetic materials is their impact strength which represents the resistance of materials to dynamic loads occurring in practice, e.g., during a prosthesis’ fall. The amount of energy absorbed by materials before they are fractured is evaluated using the Charpy or Izod tests. The values recorded with each of these two methods differed for the same materials; however, good correlations among them have been found [79,80]. In this study, the Charpy method was used with the un-notched specimens, because the notching processes is time consuming, criticized for creating stresses in the PMMA specimens, and leads to problems with reproducibility [48,81]. A significant reduction in impact strength was obtained with increasing filler concentration. The impact values obtained for the A0 material were similar or higher than those recorded for PMMA denture base materials tested using an analogous methodology [48]. Impact strength decreased with conditioning time which corresponds well to the other results [82].

This reduction of flexural and impact strength values was related to the obtained inhomogeneous morphology and presence of aggregation, described in the previously published paper focused on antimicrobial properties and their stability [37]. The strong tendency of the inorganic submicron or nanofillers to aggregate is typical and related with their large surface area that provides high surface energy [83]. This problem may lead to decreased chemical interaction between the particles and the polymeric matrix [84]. The inhomogeneities in materials act as structural defects causing stress concentrations and strength reduction [85,86]. This corresponds well with the results of SEM observations (Figure 4 and Figure 6) which showed the changes of morphologies of fractured samples and more brittle behavior of the composites. Observed areas showing the presence of spherical shapes, determined by the shape of PMMA pre-polymerized particles of the “powder” component, indicated a significant local reduction in material strength as a consequence of filler aggregations, leading to uprooting of these particles from the cured material and probably accelerated fracture of samples. A similar decrease in the mechanical properties along with the increase in mass concentrations of fillers has been noted after the introduction of particles, e.g., ZrO_2_, nanodiamonds, Al_2_O_3_, hydroxyapatite, titanium oxide, ground fillers of natural origin or even glass fibers [83,87,88,89]. On the other hand, potential reinforcement in many cases can be achieved by using the synergistic effect of various additives (e.g., different particles, glass meshes, and glass fibers) [90].

Hardness is another important mechanical property of denture base materials. There are several methods for testing the hardness of polymer materials, but, frequently, the experiments conducted with denture base materials use Vickers microhardness test [91,92]. However, in this study, the ball indentation hardness test was used because of the morphology of materials determined by the used powder–liquid system. If one considers that the mass of the used monomer is about 27% of the mass of components, it becomes obvious that only part of the surface of the samples shows the presence of S–P particles. Moreover, the size of the used pre-polymerized PMMA particles was similar or larger than the expected size of indentation left during the Vickers microhardness test, so the risk of indentation on unmodified areas of materials can be assumed. For this reason, it was considered that making larger-sized indentations would provide more representative results. The increase in hardness after the introduction of the antimicrobial inorganic filler into the material was consistent with the results of other works, where a similar effect was reported as a result of the addition of metal oxides, mica, and glass particles [71,83,84,93]. A reduction in hardness during 90 days of storing was expected because water, like many other liquids, acts as a solvent to the acrylates which has been described as the plasticizing effect [94]. Water molecules penetrate into the material which leads to the separation of polymer chains, because molecules do not form basic chemical bonds with it but only occupy spaces and reduce interactions among chains such as secondary bonding and entanglements [95]. This process is typical, and a reduction in hardness values for acrylates or acrylate-based composites is frequently reported [95,96,97,98].

Higher hardness values are usually correlated with wear resistance [83,99,100]. However, for inhomogeneous materials, selective damage during wear may occur, so the hardness measurements are not sufficient to determine how the material will behave in this respect. The wear resistance of acrylate-based materials is usually considered in the context of using denture teeth [101,102,103] or restorative composites [104,105,106]. However, denture base materials should also present sufficient abrasion resistance to avoid wear by food, abrasive denture cleansers [76] or other functional forces created, for example, by the tongue [107,108]. The introduction of inorganic filler allowed to reduce abrasion with increasing S–P concentration, even by 95%, and allowed to gradually change the intensity of the occurrence of scratches, areas of surface delamination, areas indicating plastic deformation and fatigue wear mechanism. For samples of A5 composite stored up to 30 days, only uniform abrasion was observed with slight surface scratches in the direction of the movements of the antagonist. These changes are beneficial because they lead to a reduced risk of abrasion products such as polymer particles or fillers getting into the body, for example, with saliva or foods. Interestingly, this problem and its potential long-term consequences for patients’ health have not yet been studied. The reduction in the number and size of scratches, craters, and other damage that occurs on the surface of materials during abrasion is also important due to the fact that they are potential areas where increased adherence of yeast-like fungi to the surface of materials can occur [21,23,109].

After seven days of storing in distilled water, all experimental materials showed values of sorption and solubility below the maximum limit of 32 µg/mm^3^ and 1.6 µg/mm^3^, respectively, allowed by the EN ISO 20795-1:2013-07 standard. Convergent results for PMMA denture base materials were obtained in other works for commercial and experimental materials [110,111,112,113]. Furthermore, Ergun et al. [85] reported a two-fold increase in sorption and more than three-fold increase in solubility with increasing concentration of zirconium oxide nanoparticles introduced to PMMA denture base resin. The penetration of water or aqueous solutions into the material also has an impact on its properties, because acrylates can undergo slow degradation due to the fact of hydrolysis as well as enzymatic reactions [78] which affects cytotoxicity and tribological and mechanical properties [114,115]. In this background, the lack of differences in the materials’ sorption values is favorable. The low solubility value is particularly important, because the leaching of residuals of monomers and other additives used in prosthetic materials, as well as their penetration into the organism, are considered unfavorable [116,117]. In this context, the enhanced solubility justifies future research with complimentary techniques to understand the release of specific ions and chemical compounds from the materials. Research using techniques such as ICP-OES (optical emission spectrometry in inductively coupled plasma) or ICP-MS (inductively coupled plasma mass spectrometry) may provide answers to questions related to the release of, for example, silver or zirconium ions from composites containing S–P [118,119,120]. Chromatography techniques can be used to determine the release of compounds such as the residual monomer or dibenzoyl peroxide [121,122,123]. These investigations can be considered for selected composites together with analyses for other materials filled with S–P and based on different matrixes [53,54].

To sum up, on the basis of previously published and current laboratory studies, it should be stated that promising compilations of different properties were obtained for materials from A4 to A6. Those composites showed a strong effect against *C. albicans*, over 90 days of in vitro investigation, and acceptable physicochemical properties. However, the disadvantages of introducing these concentrations of S–P were a significant reduction of flexural strength and impact strength which was caused by the presence of structured defects and the more brittle behavior of tested composites. The reduction in impact strength was particularly negative. The increase in solubility recorded for these materials was also unfavorable. Determining its exact causes requires further research. Increased wear resistance, elimination of surface delamination, plastic deformation, and fatigue wear mechanisms observed during wear tests were favorable and can partially prevent entering abrasion products into the organism. Changes in hardness were also beneficial, because they indicated increased resistance to plastic deformation under localized mechanical loads. For materials with lower filler concentrations (A1–A3), changes in physicochemical properties were smaller, but they were not outweighed by other benefits, because the antimicrobial effect was short-lived.

## 5. Conclusions

Within the limits of this study, it can be concluded that the experimental composites showed a satisfactory combination of physicochemical properties. With increasing S–P concentration after 2 day conditioning in distilled water, reduced values of flexural strength (from 107 to 72 MPa), impact strength (from 18.4 to 5.5 MPa) as well as enhanced solubility (from 0.95 to 1.49 µg/mm^3^) were reported. These changes were unfavorable, but the recorded values were at acceptable levels and also within the context of the requirements of the ISO 20795-1:2013 standard. Favorable changes included increased hardness (from 198 to 238 MPa), flexural modulus (from 2.9 to 3.3 GPa), and decreased volume loss during wear test (from 2.9 to 0.2 mm^3^). The sorption values were stable. The percentage changes of the analyzed properties during storage in distilled water were similar for all materials. Cytotoxic tests need to be performed in future experiments as well as long-term studies on the release of ions and other components, such as MMA monomer or dibenzoyl peroxide, from the materials.

## Figures and Tables

**Table 1:** Mass concentrations of the antimicrobial filler in relation to the individual components of the “powder–liquid” system.

Material Code	Concentration of Filler after Milling with Powder, %	Concentration of Filler in the Weight of The Liquid, %
A0 (Control)	0	0
A1	0.25	0.7
A2	0.5	1.3
A3	1	2.6
A4	2	5.1
A5	4	9.9
A6	8	18.6

**Table 2:** The results of one-way ANOVA and Tukey’s HSD post-hoc tests for flexural strength. *

Material Code	Storing Time /Days				
	2	7	30	60	90
	(*p* ˂ 0.0001)	(*p* ˂ 0.0001)	(*p* ˂ 0.0001)	(*p* ˂ 0.0001)	(*p* ˂ 0.0001)
A0 (*p* = 0.0076)	A; a	A; a	A; a,b	A; a,b	A; b
A1 (*p* = 0.0066)	A; a	A,B; a	A,B; a,b	A,B; a,b	A; b
A2 (*p* = 0.0062)	A,B; a	B,C; a,b	B,C; a,b	B,C; b	A,B; b
A3 (*p* = 0.0459)	B,C; a	C,D; a,b	C,D; a,b	C,D; a,b	B,C; b
A4 (*p* = 0.0427)	C,D; a	D,E; a,b	D,E; a,b	D,E; a,b	C,D; b
A5 (*p* = 0.0342)	D,E; a	E,F; a,b	E,F; a,b	E,F; a,b	D; b
A6 (*p* = 0.0084)	E; a	F; a	F; a,b	F; a,b	E; b

* The different uppercase letters (A–F) for each column and lowercase letters (a–b) for each row show significantly different results at the *p* < 0.05 level.

**Table 3:** The results of one-way ANOVA and Tukey’s HSD post-hoc tests for flexural modulus.*

Material Code	Storing Time /Days				
	2	7	30	60	90
	(*p* = 0.2185)	(*p* = 0.1563)	(*p* = 0.0447)	(*p* = 0.0237)	(*p* = 0.0076)
A0 (*p* = 0.0848)	-	-	A	A	A
A1 (*p* = 0.1101)	-	-	A,B	A,B	A,B
A2 (*p* = 0.1662)	-	-	A,B	A,B	A,B
A3 (*p* = 0.3100)	-	-	A,B,C	A,B,C	B,C
A4 (*p* = 0.6143)	-	-	A,B,C	B,C	B,C,D
A5 (*p* = 0.6612)	-	-	B,C	C	C
A6 (*p* = 0.5290)	-	-	C	C	D

* The different uppercase letters (A–D) for each column show significantly different results at the *p* < 0.05 level.

**Table 4:** The results of one-way ANOVA and Tukey’s HSD post-hoc tests for impact strength. *

Material Code	Storing Time /Days				
	2	7	30	60	90
	(*p* < 0.0001)	(*p* < 0.0001)	(*p* < 0.0001)	(*p* < 0.0001)	(*p* < 0.0001)
A0 (*p* = 0.1882)	A	A	A	A	A
A1 (*p* = 0.0084)	B; a	B; a,b	B; a,b	B; a,b	B; b
A2 (*p* = 0.1760)	B	B,C	C	B	B
A3 (*p* = 0.0017)	C,D; a	C,D; a	C; a	C; a,b	C,D; b
A4 (*p* = 0.0017)	D; a	D; a	C,D; a,b	C,D; b	D; b
A5 (*p* < 0.0001)	D; a	D; b	D; b	C,D; b	D; b
A6 (*p* < 0.0001)	D; a	D; a	D; b	D; b	D; b

* The different uppercase letters (A–D) for each column and lowercase letters (a–b) for each row show significantly different results at the p < 0.05 level.

**Table 5:** The results of one-way ANOVA and Tukey’s HSD post-hoc tests for ball indentation hardness. *

Material Code	Storing Time /Days				
	2	7	30	60	90
	(*p* < 0.0001)	(*p* < 0.0001)	(*p* < 0.0001)	(*p* < 0.0001)	(*p* < 0.0001)
A0 (*p* < 0.0001)	A; a	A; b	A; b	A; c	A; c
A1 (*p* < 0.0001)	A,B; a	B; a	A,B; a	A,B; b	A,B; b
A2 (*p* < 0.0001)	B,C; a	B,C; a	B,C; a,b	B,C; b,c	B,C; c
A3 (*p* < 0.0001)	B,C; a	C,D; a	C,D; a,b	C,D; b,c	C,D; c
A4 (*p* < 0.0001)	C,D; a	C,D; a	C,D; a,b	D,E; b,c	D; c
A5 (*p* < 0.0001)	C,D; a	D,E; a	D,E; a,b	E; b,c	D,E; c
A6 (*p* < 0.0001)	D; a	E; a,b	E; b	E; c	E; c

* The different uppercase letters (A–E) for each column and lowercase letters (a–c) for each row show significantly different results at the p < 0.05 level.

**Table 6:** The results of one-way ANOVA and Tukey’s HSD post-hoc tests for volume loss. *

Material Code	Storing Time /Days				
	2	7	30	60	90
	(*p* < 0.0001)	(*p* < 0.0001)	(*p* < 0.0001)	(*p* < 0.0001)	(*p* < 0.0001)
A0 (*p* < 0.0425)	A; a,b	A; a,b	A; a	A; b	A; c
A1 (*p* < 0.0001)	B; a	B; b	B; b	B; b	B; c
A2 (*p* < 0.0001)	C; a	C; a	C; a	B; b	B; c
A3 (*p* < 0.0001)	C,D; a	D; a	C,D; b	C; c	C; c
A4 (*p* < 0.0001)	D,E; a	D; a,b	D; b	C; b	C; c
A5 (*p* < 0.0001)	D,E; a	D; a	E; a	C; b	C; b
A6 (*p* < 0.0001)	E; a	D; b	E; b	C; c	C; c

* The different uppercase letters (A–E) for each column and lowercase letters (a–c) for each row show significantly different results at the p < 0.05 level.
